# Association between Iron Status and Incident Type 2 Diabetes: A Population-Based Cohort Study

**DOI:** 10.3390/nu12113249

**Published:** 2020-10-23

**Authors:** Andrés Díaz-López, Lucía Iglesias-Vázquez, Meritxell Pallejà-Millán, Cristina Rey Reñones, Gemma Flores Mateo, Victoria Arija

**Affiliations:** 1 Medicine and Health Sciences Faculty, Universitat Rovira i Virgili (URV), 43201 Reus, Spain; andres.diaz@iispv.cat (A.D.-L.); lucia.iglesias@urv.cat (L.I.-V.); 2 Institute of Health Research Pere Virgili (IISPV), 43204 Reus, Spain; 3 Center of Biomedical Research in Physiopathology of Obesity and Nutrition (CIBEROBN), Institute of Health Carlos III, 28029 Madrid, Spain; 4 Research Group in Nutrition and Mental Health (NUTRISAM), URV, 43201 Reus, Spain; 5 Unit of Research Support Reus-Tarragona, Jordi Gol University Institute for Primary Care Research (IDIAP), 43202 Tarragona, Spain; meritxell.palleja@urv.cat (M.P.-M.); cristina.rey@urv.cat (C.R.R.); gfloresm@xarxatecla.cat (G.F.M.)

**Keywords:** serum ferritin, iron status, type 2 diabetes, primary healthcare, type 2 diabetes incidence

## Abstract

Type 2 diabetes poses a major public health challenge. Here, we conducted a cohort study with a large sample size to determine the association of baseline serum ferritin (SF), a marker of iron status, with incident type 2 diabetes in primary healthcare patients in Catalonia, a western Mediterranean region. A total of 206,115 patients aged 35–75 years without diabetes and with available baseline SF measurements were eligible. The variables analyzed included sociodemographic characteristics, anthropometry, lifestyle, morbidity and iron status (SF, serum iron and hemoglobin). Incident type 2 diabetes during follow-up (2006–2016) was ascertained using the International Classification of Diseases, 10th edition. Cox proportional-hazards models adjusted for multiple baseline confounders/mediators were used to estimate hazard ratios (HRs). Over a median follow-up of 8.4 years, 12,371 new cases of type 2 diabetes were diagnosed, representing an incidence rate of 7.5 cases/1000 persons/year. Since at baseline, the median SF concentration was higher in subjects who developed type 2 diabetes (107.0 µg/L vs. 60.3 µg/L; *p* < 0.001), SF was considered an independent risk predictor for type 2 diabetes; the multivariable-adjusted HRs for incident type 2 diabetes across SF quartiles 1–4 were 1.00 (reference), 0.95 (95% CI = 0.85–1.06), 1.18 (95% CI = 1.65–1.31) and 1.51 (95% CI = 1.36–1.65), respectively. Our study suggested that higher baseline SF was significantly associated with an increased risk of new-onset type 2 diabetes in Catalan primary healthcare users, supporting the relevance of monitoring iron stores in order to improve the diagnosis and management of diabetes in clinical practice.

## 1. Introduction

Type 2 diabetes is a major public health concern globally due to its negative impact on quality of life, increased morbidity and mortality and growing healthcare costs [1]. Despite advances in community-based interventions, the global prevalence of type 2 diabetes among adults rose from 5.9% in 2006 to 6.28% in 2017, with projections of reaching 7.08% by 2030 if current trends continue [2]. Effective public health and clinical preventive measures are urgently needed.

Iron overload has been described as a possible cause of type 2 diabetes and its comorbidities, and various iron-related biomarkers have been repeatedly associated with type 2 diabetes [3,4]. Several prospective and case-control studies have examined the association between serum ferritin (SF), the most commonly used indicator of body iron stores, and the risk of type 2 diabetes [5]. The results are conflicting, with studies showing a positive association between SF levels and type 2 diabetes [4,6,7,8], while others fail to corroborate this association in both sexes [9,10,11], in men [11] and in women [12,13]. Furthermore, in some studies reporting sex differences, the direction of these differences was not consistent across studies [9,10,11,12,13]. The reasons for these contrasting findings might correspond to differences in recruitment, methods and SF measurements (serum or plasma samples), and the short follow-up and criteria for the diagnosis of type 2 diabetes.

In addition, comorbidities, inflammatory statuses and unhealthy lifestyles can increase the risk of iron overload, especially among populations with genetic risk factors. For instance, Europe has a distinctive geographical distribution for the most common mutations in the hemochromatosis-associated HFE gene, C282Y and H63D [14]. In this respect, some regions of Spain have reported an H63D mutation prevalence of up to 46% [15]. Moreover, dietary and lifestyle habits can significantly differ between the Mediterranean area and other European regions [16].

Epidemiological data on the association of iron status and type 2 diabetes involving southern European populations remain insufficient [17]. There is a need for studies involving large medical records databases to establish the association between SF and type 2 diabetes at a population scale. Notably, most prior studies have focused on only one sex, or sex-stratified analyses were often not reported. We conducted a large-scale cohort study to investigate the longitudinal effects of baseline SF levels on incident type 2 diabetes in primary healthcare patients aged between 35 and 75 years in Catalonia, a western Mediterranean region. We also performed stratified analyses to explore the potential heterogeneity among different subgroups.

## 2. Materials and Methods

### 2.1. Study Design

This was a longitudinal study using data from extensive population databases of adult primary healthcare users in Catalonia, a Mediterranean region located in north-eastern Spain. The study population included patients between 35 and 75 years of age, who at baseline (1 January 2006–31 December 2008), had registered SF values and no clinical diagnosis of type 2 diabetes (inclusion criteria). Exclusion criteria: patients with a clinical diagnosis of type 2 diabetes (International Classification of Diseases, 10th edition (ICD-10): codes E11–E14) or using antidiabetic medication (oral or insulin), a history of illegal drug use, chronic alcoholism (or a total daily alcohol intake > 50 g), haemochromatosis, chronic conditions (such as liver, rheumatic and kidney disease) and acute infection or inflammation. Furthermore, individuals institutionalized, using iron supplements, reporting high C-reactive protein (CRP) levels, having low hemoglobin (Hb) concentrations (<10 g/dL), having a mean corpuscular volume (MCV) < 80 or having any other altered iron-related biomarker were also excluded from the study.

### 2.2. Data Sources

The data for this study were extracted from the Information System for Research in Primary Care (SIDIAP database, www.sidiap.org) for the years 2006 to 2016, inclusive. Briefly, the SIDIAP is a clinical computerized database containing anonymized longitudinal information of about six million patients (>80% of the Catalan population, 15% of the Spanish population). The data are recorded by health professionals (general practitioners and nurses) during routine visits using a specific piece of software called eCAP, implemented in all Primary Care Centers (PCC) of the Catalan Health Institute, the larger health provider in Catalonia, directly operating 287 primary healthcare centers. The information recorded includes sociodemographic characteristics, clinical and lifestyle factors (i.e., body mass index (BMI), smoking status and alcohol use), morbidity (ICD-10 codes), specialist physician reports, and laboratory tests. Information on prescribed drugs was provided by the CatSalut general database. The quality of the SIDIAP database has been previously validated [18].

The study was approved by the Ethics Committee of the Primary Health Care University Research Institute (IDIAP) Jordi Gol. The study complies with the tenets of the Declaration of Helsinki.

### 2.3. Incidence of Type 2 Diabetes

The primary outcome was the incidence of type 2 diabetes (ICD-10: E11–E14). Patients without baseline type 2 diabetes, registered in the SIDIAP database at recruitment, who met at least one of the following criteria in subsequent check-ups were considered as incident (new-onset) cases of type 2 diabetes: fasting plasma glucose ≥ 7 mmol/L, 2 h plasma glucose ≥ 11.1 mmol/L, or glycated hemoglobin (HbA1c) ≥ 6.5% (≥47.5 nmol/mol).

### 2.4. Clinical and Biochemical Variables

The following information, defined a priori, was recorded at baseline and available for each patient: sociodemographic data including age, sex and BMI; lifestyle and toxic habits including smoking status and the risk of alcoholism, which was measured with Alcohol Use Disorders Identification (AUDIT-C, total scores ranging from 0 to 12) test [19] and categorized as no risk (0 scores), low risk (scores of 1–3), moderate risk (scores of 4–9) and high risk (scores of ≥10); diagnoses of hypertension (ICD10: I10–I13) and/or dyslipidemia (E78.x code). Biochemical determinations of hematocrit, MCV, Hb, SF, serum iron and CRP levels were performed. SF levels, the independent variable for this study, were measured by immunoturbidimetry (intra- and inter-assay coefficients of variation < 8).

### 2.5. Statistical Analysis

Statistical analyses were performed using STATA, version 15.0 (StataCorp LP, College Station, TX, USA). Descriptive data are presented as mean (SD), median (IQR) or numbers (%). The baseline characteristics of the study population with respect to incident type 2 diabetes were compared using Student’s t-test or the Mann–Whitney U test for continuous variables and the chi-square test for categorical variables, as appropriate. Comparisons according to SF sex-specific quartiles (Q) were performed using ANOVA and chi-square tests, as appropriate. Since SF was left-skewed, it was log-transformed prior to analyses. Frequency histograms were used to visualize the distribution of SF levels in women and men.

Cox proportional-hazards regression models were used to estimate the hazard ratios (HRs) and 95% confidence intervals (CIs) for incident type 2 diabetes according to baseline SF quartiles (with the lowest quartile as the reference) and for each SD increase in log-transformed SF concentration. Besides the unadjusted model, 4 other models with additional adjustment for key potential confounders or mediators were fitted: Model 1 was adjusted for age (years, continuous) and sex; Model 2 was additionally adjusted for smoking (smoker, non-smoker or ex-smoker), alcoholism risk score (0, 1, or 2 and 3), hypertension or hypertensive treatment (yes or no) and dyslipidemia or cholesterol-lowering treatment (yes or no); Model 3 was additionally adjusted for BMI (kg/m^2^, continuous); and Model 4 was additionally adjusted for CRP (mg/L, log-transformed continuous). The multivariable-adjusted cumulative incidence rate of type 2 diabetes plotted according to SF quartile was estimated by the Kaplan–Meier method, with the log-rank test used for comparisons.

Since men had significantly higher SF concentrations than women, we stratified the results by sex. We, therefore, show stratified analyses in pre-specified subgroups according to age (<50 or ≥50 years), smoking status (non-smoker or smoker/ex-smoker), alcoholism risk score (0 or ≥1), obesity (yes, ≥30 kg/m^2^, or no, <30 kg/m^2^), hypertension (yes or no), dyslipidemia (yes or no) and CRP (<3 (median) or ≥3 mg/L) for each sex. In this case, the corresponding multivariable-adjusted HR (95% CI) for incident diabetes associated with a 1 SD increase in log-transformed SF was provided.

In order to assess the robustness of our main results, we conducted sensitivity analyses by rerunning all the models (1) excluding participants who developed type 2 diabetes within the first 2 years of follow-up to avoid reverse causation; (2) excluding participants with baseline SF three times higher than the SD of the mean (SF ≥ 821.2 µg/L for men and ≥300.5 µg/L for women); (3) truncating outliers of SF outside the 1st (6 µg/L) to 99th (642.8 µg/L) percentiles of the distribution to minimize unduly influence by outliers and to improve precision; and (4) assessing SF within the range most strongly and linearly correlated with iron stores (20–300 µg/L inclusive) instead of the full range of SF. The obvious disadvantage is that the sample size for different adjusted models could be substantially diminished by a moderate proportion of missing data. Thus, we also repeated the analyses using multivariable multiple imputation by the Markov chain Monte Carlo method (5 copies) to estimate missing data for BMI (14.9%), alcoholism risk score (18.2%), smoking (41.8%) and CRP (60.5%). The imputation model included all the covariates considered in the full adjusted model (Model 4). Estimates were combined using Rubin’s rule. A 2-sided *p* < 0.05 was considered statistically significant.

## 3. Results

A total of 206,115 participants with baseline SF measurements were included in the final analyses (85.2% of the total study population). The majority of the participants were healthy middle-aged women (mean age, 50.9 years; 72% women) with normal levels of iron-related biomarkers. Most baseline characteristics were similar in patients included and excluded from the study. During a median follow-up of 8.4 (7.6–9.1) years (2006–2016), 12,371 (6%) incident type 2 diabetes diagnoses were identified in the SIDIAP database, which corresponds to a crude incidence rate of 7.5 cases per 1000 persons/year.

Table 1 shows that compared with non-diabetic subjects, patients with new-onset type 2 diabetes were older; had a higher BMI; were more likely to present comorbidities such as obesity, hypertension and dyslipidemia; and had higher SF concentrations (all *p* < 0.001).

The baseline characteristics of the study subjects according to sex-specific quartiles of SF concentrations are shown in Table 2. Participants in the highest SF quartile were older, were less likely to be current smokers, had a higher risk of alcoholism, had a higher prevalence of hypertension and dyslipidemia, and had a higher BMI than those in the lower quartile (all *P*_trend_ < 0.001). They also had higher concentrations of serum iron, Hb and CRP, and higher hematocrit and MCV (all *P*_trend_ < 0.001).

**Figure 1:**
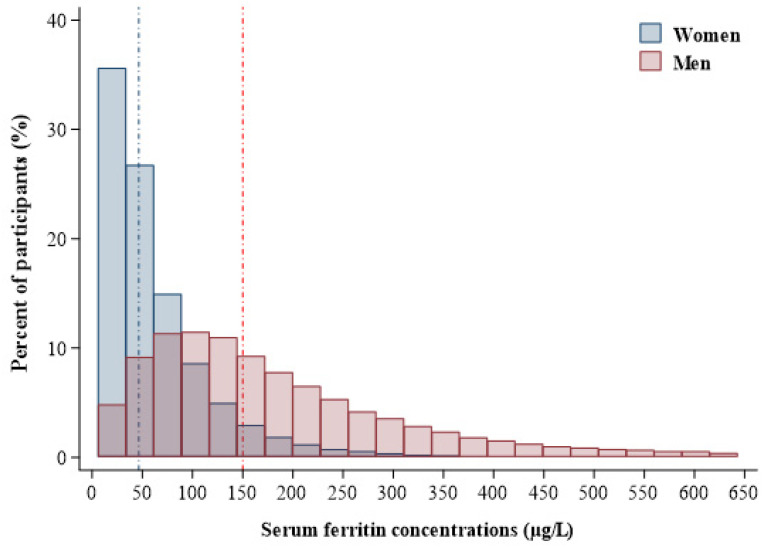
Frequency distribution of serum ferritin concentrations in men (red area) and women (blue area). Vertical lines represent the medians of serum ferritin levels in men (red line; serum ferritin = 150 µg/L) and women (blue line; serum ferritin = 46.6 µg/L), and the box is truncated at the 1st (6 µg/L) and 99th (642.8 µg/L) percentiles.

The SF concentrations in men and women, truncating outliers of SF outside the 1st (6 µg/L) to 99th (642.8 µg/L) percentiles, are shown in Figure 1. The median SF concentrations were 46.6 µg/L (IQR, 25.5–83.1 µg/L) and 150.0 µg/L (IQR, 88.0–244.0 µg/L) in women and men, respectively.

**Figure 2:**
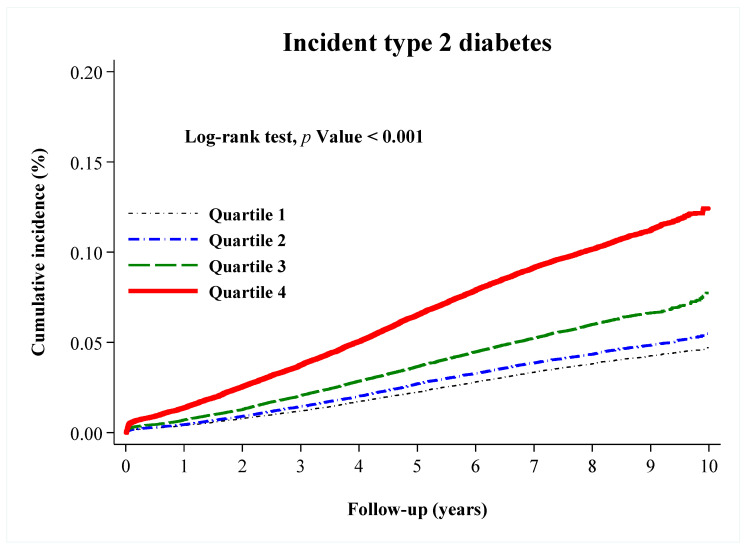
Kaplan–Meier curves showing the adjusted cumulative incidence of type 2 diabetes according to sex-specific quartiles (Q) of serum ferritin: Q1 (≤90.0 in men; ≤25.0 in women) (black dashed line), Q2 (90.1–154.6 in men; 25.1–46.0 in women) (blue dashed line), Q3 (154.7–258.0 in men; 46.1–82.7 in women) (green dashed line), and Q4 (≥258.1 in men; ≥82.8 in women) (red line). *p* values were for the overall comparison among groups according to the log-rank test.

The unadjusted incidence rate of diabetes rose across increasing quartiles of baseline SF; the incidence in the upper quartile (9.9%) was approximately 2.5 times greater than that in the lowest quartile (3.8%). The Kaplan–Meier curve showed that participants in the top quartile of SF (≥258.1 in men; ≥82.8 in women) had the highest cumulative incidence rate of type 2 diabetes, with an apparent dose-response (log-rank *p* < 0.001) (Figure 2).

In an unadjusted Cox regression model, we found a graded relationship across increasing SF quartiles correlating with a higher risk of incident type 2 diabetes (Q4 vs. Q1: HR = 2.68, 95% CI = 2.54–2.82, *p* < 0.001; Q3 vs. Q1: HR = 1.57, 95% CI = 1.49–1.66, *p* < 0.001; Q2 vs. Q1: HR = 1.15, 95% CI = 1.08–1.22, *p* < 0.001; all *P*_trend_ < 0.001) (Table 3). Although the magnitude of the effects was slightly attenuated, the association remained robust after simultaneous adjustment for diabetes risk factors and inflammation (Model 4) (Q4 vs. Q1: HR = 1.51, 95% CI = 1.36–1.65, *p* < 0.001; Q3 vs. Q1: HR = 1.18, 95% CI = 1.65–1.31, *p* = 0.001; Q2 vs. Q1: HR = 0.95, 95% CI = 0.85–1.06, *p* = 0.370; all *P*_trend_ < 0.001). Similarly, each one-SD-higher log-transformed SF was associated with 67% (HR = 1.67, 95% CI = 1.64–1.71) and 26% (HR = 1.26, 95% CI = 1.21–1.32) increases in the risk of type 2 diabetes in the unadjusted and fully adjusted models, respectively.

**Figure 3:**
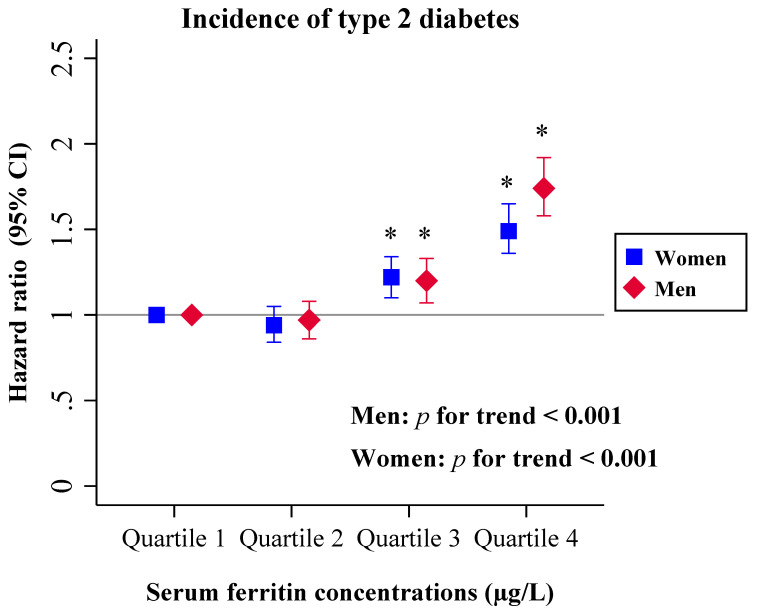
Cox proportional-hazards regression models were used to calculate HRs (95% CIs) for incidence of type 2 diabetes, according to the quartiles of serum ferritin (µg/L), separately for men (red diamond) and women (blue square). Serum ferritin quartiles (Q): Q1 (≤90.0 in men; ≤25.0 in women) (reference), Q2 (90.1–154.6 in men; 25.1–46.0 in women), Q3 (154.7–258.0 in men; 46.1–82.7 in women), and Q4 (≥258.1 in men; ≥82.8 in women). The Cox proportional-hazards regression models were adjusted for age (years), smoking (smoker, non-smoker or ex-smoker), risk levels for alcoholism (0, 1, or 2 and 3), dyslipidemia (yes or no), hypertension (yes or no) and body mass index (kg/m^2^). The horizontal line represents hazard ratio = 1. * *p* < 0.05 versus 1st quartile of serum ferritin concentration. HR, hazard ratio; CI, confidence interval.

This trend was observed in both sexes (Figure 3). When stratified by sex, the multivariable-adjusted HRs in the highest vs. lowest quartile of SF were 1.49 (95% CI = 1.36–1.65, *P*_trend_ < 0.001) for women and 1.74 (95% CI = 1.80–1.92, *P*_trend_ < 0.001) for men (*p* value for interaction = 0.037).

**Figure 4:**
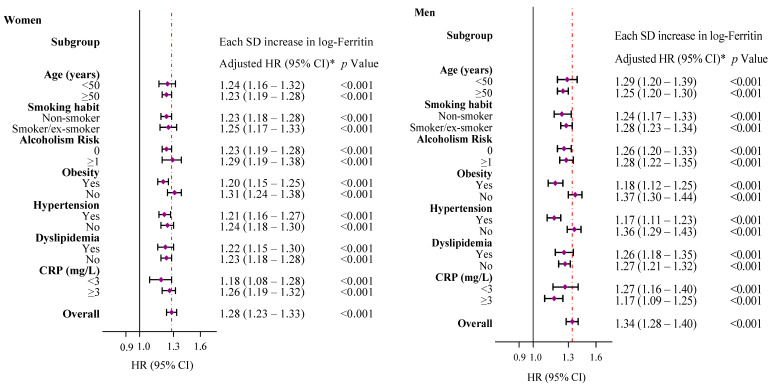
Cox proportional-hazards regression models were used to calculate HRs (95% CIs) for type 2 diabetes associated with 1-SD increase in log-ferritin for selected subgroups, separately for men and women. * Models were adjusted for age (years), smoking (smoker, non-smoker or ex-smoker), risk levels for alcoholism (0 or ≥1), dyslipidemia (yes or no), hypertension (yes or no) and obesity (yes or no), except for the variables used as a subgroup variable in each case. C-reactive protein was missing in 124,766 (60.5%) observations, and the variable was discarded from further analysis. The diamonds represent HRs, and the whisker plots represent 95% CIs. The vertical lines (red) represent the overall HRs in men and women. HR, hazard ratio; CI, confidence interval; SD, standard deviation; CRP, C-reactive protein.

Figure 4 shows that subgroup analyses by sex further confirmed the observed association between the SF concentration and type 2 diabetes risk in all categories (all *p* < 0.001). In accordance with previous studies, age ≥ 50 years (HR = 1.39, 95% CI = 1.28–1.52), smoking (HR = 1.24, 95% CI = 1.15–1.35), obesity (BMI ≥ 30 kg/m^2^, HR = 2.36, 95% CI = 2.20–2.52), hypertension (HR = 1.55, 95% CI = 1.44–1.66), dyslipidemia (HR = 1.46, 95% CI = 1.36–1.58) and an inflammatory status (CRP ≥ 3 mg/L, HR = 1.61, 95% CI = 1.49–1.72) at baseline were markedly associated with type 2 diabetes (all *p* < 0.001).

Finally, we performed sensitivity analyses under different scenarios, with no significant changes observed in the association between SF and the risk of incident type 2 diabetes (Table 4).

## 4. Discussion

This study was based on data from 206,115 primary healthcare users between 35 and 75 years of age followed up over 8.4 years. The results confirm the association between higher body iron stores (as indicated by elevated SF levels) and an increased risk of type 2 diabetes in both sexes. This association was independent of well-established risk factors such as age, toxic habits, comorbidities and inflammatory status.

To the best of our knowledge, this is the first large-scale study investigating the association between excess iron and type 2 diabetes incidence in Europe. The study participants had an average age of 50.9 years, 19.6% were smokers, and around 25% consumed alcohol regularly; a large proportion were women (72.5%), and the prevalence of obesity (26.8%), hypertension (21.5%) and dyslipidemia (13.6%) was similar to that reported in the general population [20]. We would also like to alert policy makers to the sex disparities regarding the use of health services, as underscored by the high proportion of women in our study.

The SF concentrations observed (mean: 105.2 μg/L) coincided with those found in our geographical area in the PREDIMED study population at high risk of cardiovascular disease, without type 2 diabetes at the start [17]. Similarly, the SF levels in our sample agreed with those obtained in the general population and control groups from other studies conducted in industrialized economies assessing predictors of diabetes [7,21,22,23]. Additionally, in agreement with previous European studies [7,17,22,23], the median SF concentration was higher in men (150 μg/L) than in women (46.6 μg/L).

The cumulative incidence rate of type 2 diabetes in 8.4 years of follow-up (7.5%) was similar to previous findings in Spain (6.5%) [24] and other European countries [25,26]. Importantly, the Kaplan–Meier curves show how the incidence rate of type 2 diabetes significantly increased with the SF quartiles, with results of 13.5%, 8.2%, 4.94% and 3.8% in the fourth, third, second and first quartiles, respectively. After adjusting for possible confounders including inflammation, the risk of new-onset type 2 diabetes over the 8.4-year period increased by about 50% in subjects with high SF levels, with relative risks of 1.49 for women and 1.74 for men in the highest (SF ≥ 82.8 and ≥ 258.1 µg/L, respectively) vs. lowest (SF ≤ 25 and ≤ 90 µg/L, respectively) SF quartiles. The sex differences might be partly explained by factors influencing insulin resistance such as higher visceral and hepatic adiposity and lower adiponectin levels in men, and the favorable effect of female sexual hormones such as estrogens in women [27]. Furthermore, the presence of intrinsic sexual dimorphisms at the molecular and cellular levels and sex hormones, such as testosterone and estrogen, may be responsible for these sex differences. In fact, serum ferritin levels are regulated by hepcidin, which plays a role in reducing intestinal iron absorption, and hepcidin is, in turn, associated with the mentioned sexual hormones [28]. Other factors such as menstrual blood loss or an accelerated reduction in estrogens caused by menopause could be responsible, at least in part, for the sex-related differences in ferritin levels [29]. Further research should address sex differences in iron-dependent and independent mechanisms of insulin resistance.

The associations between SF and type 2 diabetes remained robust in various sensitivity analyses, even when analyzing SF within the range that is most strongly and linearly correlated with iron stores (20–300 µg/L inclusive) and/or after stratifying by multiple study characteristics. Furthermore, this relationship persisted in both the absence and presence of known comorbidities, which may be masking hyperferritinemia. Notably, we observed a marked increase in the risk of type 2 diabetes at mildly elevated SF levels within the normal range, around 61 µg/L and 198 µg/L, the medians of the third quartiles of SF in women and men, respectively. This suggests that keeping SF concentrations below these cut-off values might reduce the incidence of type 2 diabetes and supports the relevance of monitoring iron stores.

Although some studies did not find significant differences in SF concentrations between participants with and without type 2 diabetes [30], extensive evidence from prospective research supports our observations [6,8,9,11,13,17,31], specifically, the four main current European studies analyzing this association: the EPIC-Potsdam study, a nested case-cohort study evaluating 27,548 individuals from different European countries over 7 years [8]; the Kuopio Ischemic Heart Disease Risk Factor study, a Finnish prospective cohort involving 1613 adult men with a follow-up of 16.8 years [31]; a prospective nested case-control study from the PREDIMED cohort involving 459 Spanish participants, including some from Catalonia, at high risk of cardiovascular disease who were followed-up for 6 years [17]; and the German SHIP study, which analyzed data from 3232 participants for more than 10 years [11]. All these studies found a positive correlation between SF concentrations and the risk of type 2 diabetes. Similar findings were obtained in American studies, including The Atherosclerosis Risk in Communities (ARIC) Study involving 15,792 participants [9]; a large prospective case-control study nested in the Nurses’ Health Study cohort, which assessed 1414 women for 10 years [6]; and the Aerobic Center Longitudinal Study (ACLS), which includes over 5500 participants followed over 6 years [13]. Our findings also agree with three meta-analyses: Kunutsor et al., 2013 [32], and Orban et al., 2014 [33], reported pooled relative risks in the highest vs. lowest SF quartiles of 1.73 (95% CI = 1.35–2.22) and 1.49 (95% CI = 1.19–1.86), respectively; and Jiang et al., 2019 [5], found a relative risk of type 2 diabetes of 1.22 (95% CI = 1.14–1.31) for each 100 μg/L increase in SF, which rose to 1.53 (95% CI = 1.29–1.82) when only women were considered.

While the physiopathology underlying the association between SF and type 2 diabetes remains unclear, the pro-oxidant role of iron is emphasized, since an oxidative environment can contribute to the development of insulin resistance, an increase in the HOMA index –which estimates that insulin resistance– and the dysfunction of β-cells [17,34]. Additionally, a pro-oxidant environment can activate stress pathways related to the activity of serine/threonine kinase proteins, ultimately disrupting the insulin signaling process [35].

On the other hand, the increased incidence of type 2 diabetes has been associated with other sociodemographic and lifestyle characteristics such as age [36]. In our study, people over 50 years of age had an increased risk of 39%, which might correspond to the decreased insulin sensitivity associated with an age-related decrease in B-cell proliferation capacity [36]. The age-related accumulation of abdominal and visceral fat might also contribute to insulin resistance [36,37]. Actually, our findings agree that obesity might double the risk of type 2 diabetes [38,39]. In addition to the visceral fat theory, the higher risk of type 2 diabetes in obesity could be explained by the increased production of cytokines, causing a chronic inflammatory state and mitochondrial dysfunction that could ultimately decrease insulin sensitivity and compromise the function of β-cells [39]. Further support for this hypothesis comes from the relationship observed between high CRP levels and type 2 diabetes in our study. Since SF is also an acute-phase protein that may be elevated in response to inflammatory processes, the SF–type 2 diabetes association could be the consequence of chronic sub-clinical inflammation. In contrast with other studies [40], in our data, the graded relationship between SF and type 2 diabetes remained robust after adjusting for inflammation.

Hypertension and dyslipidemia, both significant facets of metabolic syndrome, which is characterized by insulin resistance, were also statistically significant predictors of incident type 2 diabetes [41]. Our study confirmed that further adjustments for BMI, hypertension and dyslipidemia did not alter the association, suggesting that SF can predict the risk of diabetes beyond well-established risk factors.

In agreement with previous evidence, in our study, smoking was an independent predictor of type 2 diabetes, increasing its risk by 24% [42]. It has been suggested that smoking may alter iron homeostasis [43]. Consequently, it could be considered a confounding factor. However, SF levels were positively associated with diabetes after adjusting for smoking status, even after stratification by smokers and non-smokers. Further studies should elucidate the interplay between smoking, SF and type 2 diabetes.

The main strength of this study is the large sample (including 206,115 participants) we used to analyze the association between SF and type 2 diabetes. Other strengths are the follow-up of 8.4 years and the use of the SIDIAP database, which contains well-curated electronic health records (EHRs). We should also underscore several limitations inherent to observational studies that use EHRs, for instance, the risk of bias caused by the underdiagnosis of type 2 diabetes and the heterogeneity of the variables recorded by different PCC. Similarly, we cannot rule out bias due to unmeasured confounders, percentages of missing data or residual confounding. In our study, the association between SF and type 2 diabetes remained virtually unchanged before and after data imputation. Lastly, the single measurement of SF at baseline may result in random measurement errors and the attenuation of risk.

## 5. Conclusions

In conclusion, we found that elevated SF concentrations are associated with an increased risk of developing type 2 diabetes among people between 35 and 75 years of age that attend primary care services in Catalonia. We recommend the annual monitoring of iron status in order to improve the diagnosis and management of type 2 diabetes in clinical practice. Possible strategies for decreasing SF levels are the use of iron chelators, the modification of dietary patterns, reducing dietary iron bioavailability and avoiding iron-containing dietary supplements, particularly in people with elevated SF concentrations.

## Figures and Tables

**Table 1:** Baseline characteristics of study subjects based on 8.4-year new-onset type 2 diabetes status.

			Incident Type 2 Diabetes		
		Overall	No	Yes	
*n*		206,115	193,744	12,371	*p* Value ^†^
Serum ferritin (µg/L)		62.5 [30.9–127.0]	60.3 [30.0–122.0]	107.0 [53.7–207.8]	<0.001
Age (years)		50.9 ± 10.8	50.6 ± 10.8	56.5 ± 9.8	<0.001
Men, *n* (%)		56,621 (27.5)	51,579 (26.6)	5042 (40.8)	<0.001
BMI (kg/m^2^)		27.4 ± 5.1	27.2 ± 5.0	31.2 ± 5.3	<0.001
	Missing, *n* (%) *	30,848 (14.9)	30,595 (15.8)	253 (2.0)	
Smoking habit, *n* (%)					<0.001
	Smoker	40,511 (19.6)	38,059 (19.6)	2452 (19.8)	
	Non-smoker	64,503 (31.3)	60,075 (31.0)	4428 (35.8)	
	Ex-smoker	14,809 (7.2)	13,675 (7.1)	1134 (9.2)	
	Missing *	86,296 (41.8)	81,935 (42.3)	4357 (35.2)	
Alcoholism risk levels, *n* (%)					<0.001
	0	116,581 (56.6)	108,669 (56.1)	7912 (64.0)	
	1	47,896 (23.2)	44,477 (23.0)	3419 (27.6)	
	2	4113 (2.0)	3710 (1.9)	403 (3.3)	
	3	56 (0.03)	47 (0.02)	9 (0.1)	
	Missing *	37,469 (18.2)	36,841 (19.0)	628 (5.1)	
Obesity, *n* (%)		46,928 (26.8)	40.294 (24.7)	6634 (54.8)	<0.001
	Missing *	30,848 (14.9)	30,595 (15.8)	253 (2.05)	
Hypertension, *n* (%)		44,240 (21.5)	38,744 (20.0)	5496 (44.4)	<0.001
Dyslipidemia, *n* (%)		28,139 (13.6)	24,757 (12.8)	3382 (27.3)	<0.001
Serum iron (µmol/L)		84.1 ± 37.5	84.0 ± 37.5	85.6 ± 37.0	<0.001
	Missing, *n* (%) *	47,925 (23.2)	45,265 (23.4)	2660 (21.5)	
Hemoglobin (g/dL)		13.9 ± 1.3	1.9 ± 1.3	14.4 ± 1.4	<0.001
Hematocrit (%)		41.6 ± 3.7	41.5 ± 3.6	43.0 ± 3.8	<0.001
MCV (fL)		90.1 ± 4.3	90.1 ± 4.3	89.9 ± 4.5	<0.001
CRP (mg/L)		3.0 [1.4–6.0]	2.9 [1.4–5.9]	4.5 [2.4–8.9]	<0.001
	Missing, *n* (%)*	124,766 (60.5)	118,302 (61.1)	6464 (52.2)	

Data are presented as mean ± standard deviation, number (%) or median [interquartile range]. BMI, body max index; MCV, mean corpuscular volume; CRP, C-reactive protein. * The missing categories were not used in the estimation of *p* values. ^†^ *p* value was derived from an independent Student’s *t*-test (when expressed as mean), Mann–Whitney test (when expressed as median) or chi-square test (when expressed as %).

**Table 2:** Baseline characteristics of study subjects according to sex-specific quartiles of serum ferritin concentrations (*n* = 206,115).

		Quartiles (Q) of Serum Ferritin (µg/L)				
		Q1	Q2	Q3	Q4	*P*_trend_^†^
		≤90.0 in Men	90.1–154.6 in Men	154.7–258.0 in Men	≥258.1 in Men	
		≤25.0 in Women	25.1–46.0 in Women	46.1–82.7 in Women	≥82.8 in Women	
*n*		52,366	51,001	51,255	51,493	
Serum ferritin (µg/L)		19.0 [13.0–25.0]	39.0 [31.8–96.0]	68.2 [56.0–162.0]	154.0 [107.0–299.5]	<0.001
Age (years)		47.3 ± 9.8	48.9 ± 10.4	51.9 ± 10.7	55.8 ± 10.3	<0.001
Men, *n* (%)		14,204 (27.1)	14,111 (27.7)	14,184 (27.7)	14,122 (27.4)	0.294
BMI (kg/m^2^)		26.5 ± 5.0	26.9 ± 5.0	27.6 ± 5.1	28.6 ± 5.1	<0.001
	Missing, *n* (%) *	9437 (18.0)	8256 (16.2)	7102 (13.9)	6053 (11.9)	
Smoking habit, *n* (%)						
	Smoker	10,816 (20.6)	10,893 (21.4)	10,073 (19.6)	8729 (17.0)	
	Non-smoker	14,198 (27.1)	14,707 (28.8)	16,540 (32.3)	19,058 (37.0)	
	Ex-smoker	3934 (7.5)	3684 (7.2)	3697 (7.2)	3494 (6.8)	
	Missing*	23,418 (44.7)	23,418 (42.6)	20,945 (40.9)	20,212 (39.3)	
Risk levels of alcoholism, *n* (%)						<0.001
	0	29,494 (56.3)	28,604 (56.1)	29,135 (56.8)	29,348 (57.0)	
	1	11,000 (21.0)	11,534 (22.6)	12,260 (23.9)	13,102 (25.4)	
	2 and 3	760 (1.4)	839 (1.6)	1032 (2.0)	1538 (3.0)	
	Missing *	11,112 (21.2)	10,024 (19.6)	8828 (17.2)	7505 (14.6)	
Obesity, *n* (%)		9102 (21.2)	9905 (23.2)	12,139 (27.5)	15,782 (34.7)	<0.001
	Missing, *n* (%) *	9437 (18.0)	8256 (16.2)	7102 (13.9)	6053 (11.9)	
Hypertension, *n* (%)		4307 (8.2)	5835 (11.4)	7718 (15.1)	10,279 (20.0)	<0.001
Dyslipidemia, *n* (%)		7733 (14.8)	8896 (17.4)	11,505 (22.4)	16,106 (31.3)	<0.001
Serum iron (µmol/L)		74.7 ± 37.8	84.3 ± 36.2	86.1 ± 35.5	91.9 ± 38.1	<0.001
	Missing, *n* (%) *	11,363 (21.7)	12,091 (23.7)	12,625 (24.7)	11,846 (23.2)	
Hemoglobin (g/dL)		13.6 ± 1.3	13.9 ± 1.3	14.1 ± 1.2	14.2 ± 1.3	<0.001
Hematocrit (%)		40.7 ± 3.8	41.6 ± 3.6	41.9 ± 3.5	42.2 ± 3.4	<0.001
MCV (fL)		89.1 ± 4.3	90.1 ± 4.1	90.3 ± 4.1	91.0 ± 4.5	<0.001
CRP (mg/L)		2.6 [1.18–5.40]	2.9 [1.37–6.00]	3.0 [1.5–6.1]	3.4 [1.8–6.9]	<0.001
	Missing, *n* (%) *	32,917 (62.6)	31,672 (62.1)	30,518 (59.8)	29,659 (58.1)	

Data are presented as mean ± standard deviation, number (%) or median [interquartile range]. BMI, Body Mass Index; MCV, mean corpuscular volume; CRP, C-reactive protein. * The missing categories were not used in the estimation of *p* values. ^†^ *p* value for trend was derived from an ANOVA test (when expressed as mean) and chi-square test (when expressed as %).

**Table 3:** Hazard ratios (HRs) (95% CIs) for incident type 2 diabetes according to sex-specific quartiles of serum ferritin during an 8.4-year period.

	Quartiles (Q) of Serum Ferritin (µg/L)						
	Q1	Q2	Q3	Q4	*P*_trend_	Each SD (1.02 µg/L) Increase in Log-Ferritin	*p* Value
	≤90.0 in Men	90.1–154.6 in Men	154.7–258.0 in Men	≥258.1 in Men			
	≤25.0 in Women	25.1–46.0 in Women	46.1–82.7 in Women	≥82.8 in Women			
Type 2 diabetes							
*n* cases/total *n* (%)	2009/52,366 (3.8)	2237/51,001 (4.4)	3055/51,001 (6.0)	5070/51,001 (9.9)		12,371/206,115 (6.0)	
IR per 1000 person-years (%)	3.8	4.9	8.2	13.5		7.5	
Unadjusted	1.00 (Reference)	1.15 (1.08–1.22) *	1.57 (1.49–1.66) *	2.68 (2.54–2.82) *	<0.001	1.67 (1.64–1.71)	<0.001
Model 1	1.00 (Reference)	1.08 (1.02–1.15) *	1.35 (1.27–1.42) *	2.03 (1.92–2.14) *	<0.001	1.43 (1.40–1.46)	<0.001
Model 2 ^†^	1.00 (Reference)	0.98 (0.91–1.06)	1.32 (1.23–1.42) *	1.85 (1.73–1.98) *	<0.001	1.39 (1.35–1.43)	<0.001
Model 3 ^‡^	1.00 (Reference)	0.96 (0.89–1.03)	1.22 (1.14–1.31) *	1.61 (1.51–1.72) *	<0.001	1.31 (1.27–1.34)	<0.001
Model 4 ^§^	1.00 (Reference)	0.95 (0.85–1.06)	1.18 (1.65–1.31) *	1.51 (1.36–1.65) *	<0.001	1.26 (1.21–1.32)	<0.001

Cox proportional-hazards regression models were used to calculate HRs (95% CIs). HR, hazard ratio; CI, confidence interval; IR, incidence rate; SD, standard deviation. *p* value for trend based on Cox proportional-hazards regression using the median serum ferritin value within each quartile as a continuous variable. * *p* < 0.05 versus 1st quartile of serum ferritin concentration. Model 1: adjusted for age (years) and sex. Model 2: additionally adjusted for smoking (smoker, non-smoker or ex-smoker), risk levels for alcoholism (0, 1, or 2 and 3), dyslipidemia (yes or no) and hypertension (yes or no). ^†^ Total *n* = 103,114; smoking was missing in 86,296 (41.8%) observations, and the risk of alcoholism was missing in 37,469 (18.2%) observations. Model 3: additionally adjusted for body mass index (kg/m^2^, continuous variable). ^‡^ Total *n* = 96,770; body mass index was missing in 30,848 (14.9%) observations. Model 4: additionally adjusted for C-reactive protein (mg/L, log-transformed continuous variable). ^§^ Total *n* = 40,839; C-reactive protein was missing in 124,766 (60.5%) observations.

**Table 4:** Sensitivity analyses. Multivariable-adjusted HRs (95% CIs) for incident type 2 diabetes according to sex-specific quartiles of serum ferritin in various scenarios.

	Quartiles (Q) of Serum Ferritin (µg/L)						
	Q1	Q2	Q3	Q4	*P*_trend_	Each SD (1.02 µg/L) Increase in Log-Ferritin	*p* Value
	≤90.0 in Men	90.1–154.6 in Men	154.7–258.0 in Men	≥258.1 in Men			
	≤25.0 in Women	25.1–46.0 in Women	46.1–82.7 in Women	≥82.8 in Women			
Model 1	1.00 (Reference)	0.95 (0.85–1.06)	1.18 (1.65–1.31) *	1.51 (1.36–1.65) *	<0.001	1.26 (1.21–1.32)	<0.001
Model 2	1.00 (Reference)	0.93 (0.82–1.05)	1.14 (1.02–1.28) *	1.38 (1.24–1.54) *	<0.001	1.21 (1.16–1.27)	<0.001
Model 3	1.00 (Reference)	0.95 (0.85–1.07)	1.17 (1.06–1.30) *	1.44 (1.30–1.59) *	<0.001	1.23 (1.18–1.28)	<0.001
Model 4	1.00 (Reference)	0.95 (0.85–1.06)	1.16 (1.04–1.29) *	1.47 (1.33–1.63) *	<0.001	1.25 (1.21–1.31)	<0.001
Model 5	1.00 (Reference)	1.05 (0.93–1.18)	1.15 (1.03–1.29) *	1.40 (1.27–1.57) *	<0.001	1.18 (1.13–1.23)	<0.001
Model 6	1.00 (Reference)	1.04 (0.98–1.10)	1.22 (1.15–1.29) *	1.67 (1.59–1.76) *	<0.001	1.30 (1.27–1.33)	<0.001

Cox proportional-hazards regression models were used to calculate HRs (95% CIs). HR, hazard ratio; CI, confidence interval; IR, incidence rate; SD, standard deviation. *p* value for trend based on Cox proportional-hazards regression using the median serum ferritin value within each quartile as a continuous variable. All models were adjusted for age (years), sex, smoking (smoker, non-smoker or ex-smoker), risk levels for alcoholism (0, 1, or 2 and 3), dyslipidemia (yes or no), hypertension (yes or no), body mass index (kg/m^2^, continuous) and C-reactive protein (mg/L, log-transformed continuous). * *p* < 0.05 versus 1st quartile of serum ferritin concentration. Model 1: Original multivariable model. Model 2: Multivariable model excluding participants who developed type 2 diabetes within the first 2 years of follow-up (2792 events excluded). Model 3: Multivariable model excluding participants with serum ferritin levels beyond three times the SD from the mean (serum ferritin ≥ 821.2 µg/L for men and ≥ 300.5 µg/L for women). Model 4: Multivariable model truncating outliers of serum ferritin outside the 1st (6 µg/L) to 99th (642.8 µg/L) percentiles. Model 5: Multivariable model assessing serum ferritin within the range that is most strongly and linearly correlated with iron stores (20–300 µg/L inclusive) instead of the full range of serum ferritin. Model 6: Multivariable model using multiple imputation methods to estimate missing data for BMI (14.9%), risk alcoholism score (18.2%), smoking (41.8%) and C-reactive protein (60.5%).
